# Granular computing with multiple granular layers for brain big data processing

**DOI:** 10.1007/s40708-014-0001-z

**Published:** 2014-09-06

**Authors:** Guoyin Wang, Ji Xu

**Affiliations:** 1 School of Information Science & Technology, Southwest Jiaotong University, Chengdu, 610031 China; 2 Key Laboratory of Computational Intelligence, Chongqing University of Posts and Telecommunications, Chongqing, 400065 China; 3 Information Engineering School, Guizhou University of Engineering Science, Bijie, 551700 Guizhou, China

**Keywords:** Big data, Brain big data, Multi-granular computing, Data science

## Abstract

Big data is the term for a collection of datasets so huge and complex that it becomes difficult to be processed using on-hand theoretical models and technique tools. Brain big data is one of the most typical, important big data collected using powerful equipments of functional magnetic resonance imaging, multichannel electroencephalography, magnetoencephalography, Positron emission tomography, near infrared spectroscopic imaging, as well as other various devices. Granular computing with multiple granular layers, referred to as multi-granular computing (MGrC) for short hereafter, is an emerging computing paradigm of information processing, which simulates the multi-granular intelligent thinking model of human brain. It concerns the processing of complex information entities called information granules, which arise in the process of data abstraction and derivation of information and even knowledge from data. This paper analyzes three basic mechanisms of MGrC, namely granularity optimization, granularity conversion, and multi-granularity joint computation, and discusses the potential of introducing MGrC into intelligent processing of brain big data.

## Introduction of big data and data science

To gain an insight of philosophy into the nature of brain data and the significance of processing it, we would firstly introduce a broad view on some related concepts such as physical space, social space, data space, natural sciences, social sciences, and data sciences.

There have been for a long history the physical space and social space to describe the phenomena in natural world and human society, respectively, and the research on the spaces leads to natural science and social science. In recent years, the ubiquitous digitalization of both natural world and human society has produced huge amount of data. Along with “big data” becoming a hot topic for researchers, entrepreneurs and government officials, people realize that a *data space* has come into existence.

The connection and interaction among people is the one of the key sources of human intelligence; in other words, the interactions of elements in social space produce human intelligence. So, similarly, it is expected that the relations and interactions of entities in data space would produce other forms of intelligence such as machine intelligence and web intelligence [1].

The data space is “relatively independent” of physical space and social space, since it remains stable in a way despite being a reflection of them. Once the data have been generated, they will not evolve accordingly as the described objects change if no special mechanism is arranged. One dataset as a mirror of entities from natural world or human society would yield new results if interacted with others, and then the results may have reaction on natural world or human society with the assistance of automatic control devices or human beings. The data may have powerful reaction to the real world even if it is fabricated, e.g., rumors spread via mobile phones and the Internet played a vicious role in the London riot 2011 [2].

It would be agreed that research on data space will lead to *data science* which has many differences from natural and social science with respect to research objectives, research methodologies, and technologies. In some circumstances, data science can be used interchangeably with big data [3]. To get the best out of big data, funding agencies should develop shared tools for optimizing discovery and train a new breed of researchers, says Mattmann [4]. Data Science need not be always for big data; however, the fact that data are scaling up makes big data an important aspect of data science [3].

“Big data” is the most highlighted term in the past 2 years, and it can be expected with much confidence that it would continue to be popular in the next a few years for its promising utility in many fields such as commerce and business, biology, public administration, material science, and cognition in human brain, just to name a few. People from the society of academia, industry, and the open source community have done a lot of work concerning big data analytics.

The studies of big data by academia society could be classified into two categories: basic researches and application researches.

The basic researches of big data are about basic concepts, rules, procedures, and so on. Fisher discussed the challenges lying in the interactions in big data analytics [5]. A community white paper developed by leading researchers across the United States discussed the application of big data in several typical fields and proposed a data analysis pipeline [6]. Recently, Wu presented a HACE theorem that characterizes the features of the big data revolution, and proposed a 3-tiered big data processing model [7]. A close-up view about big data was demonstrated by Chen and Zhang, which included applications, opportunities, and challenges of big data; the state-of-the-art techniques and technologies; as well as several underlying methodologies to handle the data deluge [8]. Han presented a novel skyline algorithm on big data showing significant advantage over the existing skyline algorithms [9], and there are many other researches falling into this category such as [10–13].

Application researches on big data refer to the applications of big data analytics in many different fields. In commerce and business, Chen introduced in detail the evolution of business intelligence, analytics, and the impact of big data in typical areas [14]. In biology, powerful computers and numerous tools for data analysis is crucial in drug discovery and other areas, and biologists get neither their feet nor their hands wet [15]. In public administration, the Trento big data platform offers the service of representing the mean availability of cars in regions of Munich at noon, which can be easily used to improve customer satisfaction, by identifying bottlenecks [16]. In materials science, advances in data analysis have placed the field on the verge of a revolution in how researchers conduct their work, analyze properties and trends in their data, and even discover new materials [17].

There are also quite a few research works which address some challenges in big data analytics with keywords like “huge data,” “large scale dataset,” and “high speed streaming data,”, but no “big data”. These works surely should be noticed and appreciated by big data researchers and practitioners [18–20].

The international IT giants such as Google, IBM, Microsoft, Oracle, and EMC have developed their own big data solution systems and platforms, which are Dremel, InfoSphere BigInsights and InfoSphere Streams, HDInsight, ExaData, Greenplum and so forth [21–26]. Most of the big data platforms are based on Hadoop. Apache also supports other projects related to Hadoop such as HBase, Hive, Pig, Mahout, and Spark, each of which has special effect in dealing with different challenging aspects in big data processing (BDP) [27]. In addition to the projects supported by Apache, there are other open source big data projects, such as Cloudera Impala [28] and RHIPE [29].

The rest of the paper is organized in the following fashion. Section 2 discusses brain big data and its applications. Section 3 introduces the three mechanisms of MGrC and discusses their relationship with five major theoretical models of MGrC. Some key issues of BDP based on MGrC are also analyzed in this section. In Sect. 4, we propose the potential of using MGrC to explore brain big data. The conclusions are drawn in Sect. 5.

## Brain big data

Among the methods of generating data from natural world and human society, using equipments of fMRI, EEG, and MEG to collect brain data is of great concern from the interdisciplinary researchers of computing, neuroscience, and cognitive psychology [30]. Because the techniques of noninvasive studies of human brain function have been in widespread use to detect metabolic activity and neuronal activity throughout the brain of different subjects all around the world, huge amount of complex datasets are collected every day. There is no doubt that the brain data are a significant category of big data, which hold great potential to unlock mysteries of the human mind [31].

**Fig. 1:**
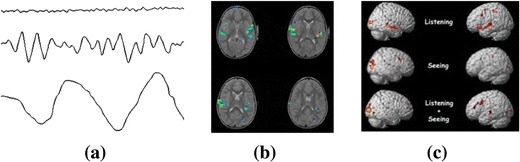
Forms of brain data. **a** Pulse curves (EEG)—http://www.trueimpact.ca/introduction-to-electroencephalogram-eeg/, **b** 2D images (fMRI)—http://irc.cchmc.org/research/fmri/cochlear.php, **c** 3D structures (reconstructed)—http://cloakunfurled.com/tag/fmri/

Brain data are in the forms of pulse curve, 2D images [32], and 3D structures reconstructed from 2D images [33], as shown in Fig. 1. Pulse curves are generated by EEG; 2D images are produced by fMRI, MEG, OCT, etc., and 3D structures are reconstructed from 2D images using computer graphics technology. Furthermore, 4D models of the brain can be based on imaging and modeling its 3D structure at a sequence of time-points [33]. We can see that brain data are more complex than regular information tables, which lead to difficulties in modeling and processing of them.

Researches on brain data can achieve a new understanding of the brain, new treatments for brain diseases (such as Alzheimer’s and Parkinson’s [34]), and new brain-like computing technologies [35]. The significance of brain data research had been realized so clearly that governments of the EU and USA started their own brain projects [35, 36]. There have been some successful researches on this field. Ryali described a novel method based on logistic regression using a combination of L1 and L2 norm regularization to identify relevant discriminative brain regions and accurately classify fMRI data [37]. Zhong and Chen proposed Data-Brain, a new conceptual model of brain data, to explicitly represent various relationships among multiple human brain data sources, with respect to all major aspects and capabilities of human information processing systems [32].

## Multi-granularity computing for big data

“GrC is a superset of the theory of fuzzy information granulation, rough set theory and interval computations, and is a subset of granular mathematics,” stated Zadeh in 1997. Granules are any subsets, classes, objects, clusters, and elements of a universe as they are drawn together by distinguishability, similarity, or functionality [38]. Yao considers GrC to be a label of theories, methodologies, techniques, and tools that make use of granules in the process of problem solving [39]. GrC has become one of the fastest growing information processing paradigms in the domain of computational intelligence and human-centric systems [38]. There are two fundamental issues in GrC: granulation and granular structure. Different semantic aspects and algorithm aspects of granulation will lead to different granular structures of the universe. Chen defined five classes of modal-style operators to construct granular structure and hierarchical structure of data based on the lattice of concepts [40].

Evolved from GrC, MGrC emphasizes jointly utilizing multiple levels of information granules (IG) in problem solving, instead of considering only one optimal granular layer.

### Three basic mechanisms and five theoretical models of MGrC

MGrC considers multiple levels of IG when solving a problem, and there have been a lot researches in this regard [41–45, 62–69]. Three basic mechanisms of MGrC can be summarized from these research works with regard to the way in which multi-granular levels are used in problem solving. They are granularity optimization, granularity conversion, and multi-granularity joint computation. In granularity optimization, the most suitable granular level of a domain is chosen for the multi-granular information/knowledge representation model (MGrR), and the most efficient and satisfactory enough solution is generated on it [41–43]. Granularity conversion means the working granularity layer will be switched between adjective layers or jump to a higher or lower granular layer, in accordance with the requirements of solving a problem [44, 45]. Multi-granularity joint computation takes a problem-oriented MGrR as input, and every layers of the MGrR are employed jointly to achieve a correct solution to the problem. Each of the three mechanisms has its particular type of problem to deal with.

The three basic mechanisms is a new perspective on GrC. Then, what is the relationship between the three mechanisms and models to implement GrC such as fuzzy set, rough set, quotient space, cloud model, and deep learning? We will see that some models suit certain mechanisms better, which are to be introduced in detail as follows.

#### Granularity optimization

The theories of fuzzy set and rough set are good choices for the mechanism of granularity optimization.

The fuzzy set theory presented by Zadeh in 1965 starts with definitions of membership function, with the more functions defined about an attribute, the attribute is granulated into the finer fuzzy IG. The reason for fuzzy IG is that crisp IG (e.g., an interval is partitioned by exact values) does not reflect the fact that the granules are fuzzy in almost all of human reasoning and concept formation [46, 47]. The number of concepts formed through fuzzy granulation reflects the corresponding granularity being relatively fine or coarse, and decision on the number is an application-specific optimization problem.

The rough set theory developed by Pawlak in 1982 is an effective model to acquire knowledge in information system with upper approximation and lower approximation as its core concepts, making decisions according to the definition of distinguishable relation and attribute reduct. Researchers of related fields have made great variety of improvements to the classic rough set theory mainly by redefining the distinguishable relation and approximation operators [48–50], and integrated it with other knowledge acquisition models, which yield rough neural computation [51], rough fuzzy set and fuzzy rough set [52], and so on.

Rough set can be used to granulate a set of objects into IGs. The grain size of the IG is determined by how many attributes and how many discrete values each attribute takes in the subset of the whole attribute set, which is selected to do the granulation. Generally, the more attributes and the more values each attribute takes, the finer the resulting IGs.

In the perspective of knowledge transformation [53], the process of data analyzing and problem solving by fuzzy sets or rough sets is actually to find a mapping from the information represented by the original finest-grained data to the knowledge hidden behind a set of optimized coarser and more abstract IGs.

#### Granularity conversion

The quotient space theory proposed by Zhang is a model for problem solving with the basic idea of conceptualizing the world at different granularities and shifting the focus of thinking onto a different abstract level [54, 55]. It is not hard to tell that quotient space is meant to solve problems with need of granularity conversion. In the quotient space theory, a problem space is described by a triplet (*X*, *f*, *T*) with *X* as its domain, *f* as its attributes, and *T* its structure. Suppose R is an equivalence relation on *X*, [*X*] is a quotient set under R. Taking [*X*] as a new domain, we have a new problem space ([*X*], [*f*], [*T*]). The worlds with different granularities are represented by a set of quotient spaces. Based on the descriptions, the construction of different-grain-sized quotient spaces and problem solving on these spaces are researched [55].

The quotient space theory has attracted the attention of researchers from the fields of information science, automatic control, and applied mathematics [56, 57]. Integrating the idea of fuzzy mathematics into quotient space theory, Zhang proposed fuzzy quotient space theory subsequently, which provides a powerful mathematical model and tool for GrC [58, 59]. Fuzzy quotient space theory introduces fuzzy equivalence relation into the construction of quotient space, in which different threshold values of the membership function will lead to quotient spaces of different grain sizes. By setting different threshold values, an MGrR can be derived.

**Fig. 2:**
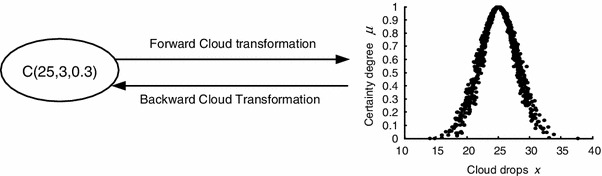
Transforming between the concept of “yong” C (*Ex* = 25, *En* = 3, *He* = 0.3) and cloud drops

The cloud model proposed by Li realizes the uncertain transformation between qualitative concepts and quantitative values and can be further used to realize the bidirectional cognition, i.e., from concept intension to extension and from extension to intension [60], as shown in Fig. 2. Since a concept definitely has the property of granularity, mapping of quantitative values to a suitable grain-sized qualitative concept is also the process of granularity optimization.

**Fig. 3:**
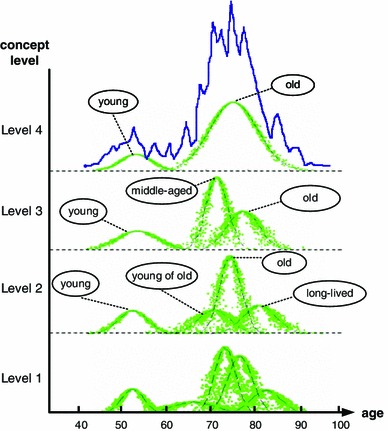
MGrR generated by A-GCT in the experiment of ACAE [61]

Inspired by the idea of MGrC, Liu constructed an MGrR using cloud model with an Adaptive Gaussian Cloud Transformation (A-GCT) algorithm [61]. Multi-granular concepts are generated by clustering academicians of Chinese Academy of Engineering (ACAE) with regard to age based on the definition of parameter *concept clarity*, as shown in Fig. 3.

Therefore, granularity conversion can be implemented using cloud model with A-GCT algorithm and a set of different values of parameter *concept clarity.*

#### Multi-granularity joint computation

**Fig. 4:**
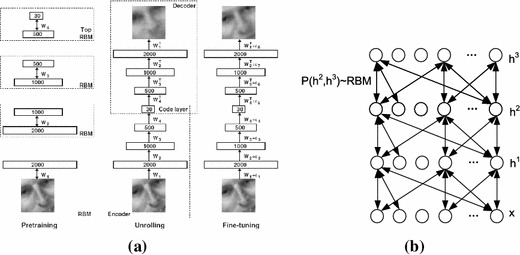
Example of RBMs and graphic model of a deep belief network based on RBMs. **a** Pretraining consists of learning a stack of RBMs, each having only one layer of feature detectors [62] and **b** graphical model of a deep belief network with observed vector **x** and hidden layers *h*^1^, *h*^2^ and *h*^3^ [63]

Deep learning is a breakthrough of learning by neural networks in recent years. Starting with Hiton publishing his research on Science magazine in 2006, whose major contribution is using deep auto-encoder networks to learn low-dimensional codes for data of high dimensionality [62], other research works of closely related topics are reported afterward [63–65]. The structure of restricted Boltzmann machines (RBMs) and a deep belief network based on RBMs are shown in Fig. 4. Deep learning also draws the attention from IT industry. Researchers from Google and Stanford University consider the problem of building high-level, class-specific feature detectors from only unlabeled data, and train a face detector on a cluster with 1,000 machines (16,000 cores) for three days, without having to label images as containing a face or not. The experiment obtains 15.8 % accuracy, a leap of 70 % relative improvement over the previous state-of-the-art [66]. Deep learning was selected as the first of the 10 breakthrough technologies 2013 [67].

**Fig. 5:**
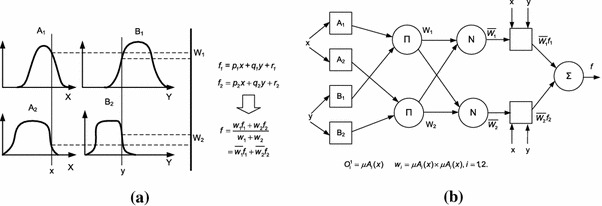
Type-3 fuzzy reasoning and its equivalent ANFIS architecture [68]. **a** Type-3 fuzzy reasoning and **b** equivalent ANFIS (type-3 ANFIS)

**Fig. 6:**
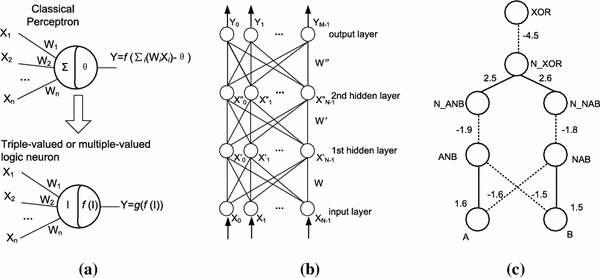
Triple-valued or multiple-valued logic neural network [70].**a** Triple-valued or multiplevalued logic neuron (TMLN), **b** multiple-layer TMLNN and **c** TMLNN for multiple-valued logic “exclusive or"

The core idea of deep learning is training a deep architecture with many layers of neural network, and the constraints between the adjacent layers are set beforehand. Although deep learning does not make the most popular topic in machine learning until 2006, the research with the similar idea can be traced back to 1990s, for example, Jang presented an adaptive-network-based fuzzy inference system (ANFIS) to implement fuzzy inference with a 5-layered neural network [68], as depicted in Fig. 5. Using a hybrid learning procedure, ANFIS can construct an input–output mapping based on both human knowledge (in the form of fuzzy if–then rules) and stipulated input–output data pairs. And we proposed a triple-valued or multiple-valued logic neural network (TMLNN) to represent and process triple-valued or multiple-valued logic knowledge using neural network [69], as illustrated in Fig. 6. The fundamental element of TMLNN is a novel neuron model, triple-valued or multiple-valued logic neuron (TMLN). Each TMLN can represent a triple-valued or multiple-valued logic rule by itself. There are two triple-valued or multiple-valued logic neurons: TMLN-AND (triple-valued or multiple-valued “logic and” neuron) and TMLN-OR (triple-valued or multiple-valued “logic or” neuron).

**Fig. 7:**
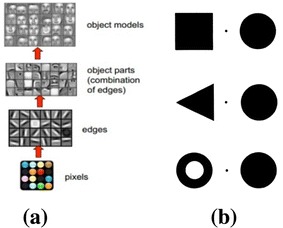
Topologically “global first” visual perception theory and deep learning. **a** Human visual perception work flow in deep learning perspective [70] and **b** the visual system was more sensitive to the topological distinction between a connected component with a hole and one with no hole [71]

The application of deep learning to pattern recognition reflects that human firstly takes as input the pixels of an object projected onto the retina and then detects the edges of the object, recognizes the parts of it, and finally forms the high-level abstract concept of the object [70], as shown in Fig. 7a. This means that visual concept formation procedure is local-to-global, which seems to be contradictory to the topologically global first visual perception theory developed by Chen in 1982 [71]. According to the results of experiments conducted by Chen’s team, the human visual system is sensitive to global topological properties, as illustrated in Fig. 7b.

However, after a careful analysis, we realize that the two theories are NOT contradictory. In fact, they reflect the different facets of human visual cognition. Chen’s theory focuses on the last phase of the whole visual concept formation, since the experiments are conducted using noninvasive measurement on human brain cortex. However, visual concept formation in deep learning considers all the organs of visual system and the whole perception process.

What can be learnt from Chen’s global first theory and deep learning from the standpoint of MGrC for BDP is that original finest-grained data (compared to the pixels projected on retina) are the information source for sure, but we should not stick to it. Exploiting components of higher level abstraction (compared to edges and parts) and the relation among them (compared to the topological relations of visual stimuli) is helpful to efficient problem solving.

Deep learning itself is a typical model of multi-granularity joint computation, and it could be expanded to a more general structure for multi-granularity joint computation (MGrJC). Major differences between MGrJC and deep learning are that the input of deep learning is the finest-grained data when MGrJC takes an MGrR as its input, and a layer-wise learner of deep learning is usually a neural network when MGrJC intends to generalize it to any type of learning model.

Although we introduce fuzzy set and rough set in section granularity optimization, and quotient space and cloud model in section granularity conversion, this does not mean that the GrC models are limited to the corresponding mechanisms. Actually, fuzzy set and rough set could be used in granularity conversion, and quotient space and cloud model could be used in granularity optimization as well. The relationship between the three mechanisms and five models is summarized in Table 1.

**Table 1:** Applicability of the MGrC models to the three MGrC mechanisms

	Fuzzy set	Rough set	Quotient space	Cloud model	Deep learning
Granularity optimization	Good	Good	OK	OK	No
Granularity conversion	OK	OK	Good	OK	No
Multi-granularity joint computation	OK	OK	OK	OK	Good

### Key issues for BDP

There are quite a few issues that remain unaddressed despite much effort having been made to BDP, among which some are caused by the same reason: always getting start analytics from the original or finest-grained data.

#### Issue 1: Lacking BDP models of human level machine intelligence (HLMI)

The founder of Fuzzy Set theory, Zadeh argues that the precisiated natural language computing, which originated from CW (computing with words), is the cornerstone of HLMI [72]. The current BDP models fail to simulate human thinking to grasp the proper granularity of information when solving a problem, and thus consequently lose the opportunity to build human-centric data processing systems. The research team led by Chen founded the topologically “global first” visual perception theory in 1982 [71]. Always dealing data from the finest granularity does not accord with this human perception law.

#### Issue 2: Lacking measures to effectively reduce the size of data in BDP

Volume is the most highlighted challenge when compared to other aspects in BDP, and many difficulties are directly caused by it. To cope with this problem, a straightforward idea is reducing the data size but preserving as much as possible its information, which could avoid excessive reliance on the finest-grained data and reduce the cost in storage and communication.

#### Issue 3: Lacking the methods to offer effective solution to big data problems with various constraints

There are some situations where a user does not insist on precise answer to a particular problem regarding BDP, since a coarser-grained imprecise result would make him/her happy enough. There are other situations where the precise answer is not available in time due to the problem complexity, data amount and complexity, and the capacity of computing and communication, but if the problem granularity is shifted to a coarser granular level, an imprecise yet acceptable result may be obtained in time. Therefore, it is necessary to introduce a term “*effective solution*”, which means that the solution meets the requirements of the user regarding granularity and timeliness simultaneously, or in other words, that the solution has a fine enough granularity with respect to the user’s quest and it is delivered in time.

MGrC are able to tackle the issues listed above. For Issue 1, computation with information described in natural language ultimately reduces to computation with granular values, which is the province of GrC [72]. Therefore, MGrC will help BDP move toward HLMI. For Issue 2, multi-granular representation of original data is a form of simplification or abstraction; hence the considerable reduction in data volume can be realized. And when it comes to Issue 3, the most highlighted feature of employing MGrC in BDP is that it can manage to offer effective solution under various constraints.

## MGrC for brain big data

As mentioned in Sect. 2, the targets for brain BDP achieve a new understanding of the brain, new treatments for brain disease, and new brain-like computing technologies. So the targets are mainly qualitative rather than quantitative, that is, we do not need a solution of precise value or mathematic function, but a result that can be described with words. This is the very province of MGrC.

There have been some related works on pulse signal processing and remote sensing images with GrC methodology, from which the future research on processing brain big data with MGrC can benefit a lot. For example, Gacek and Pedrycz developed a general framework of a granular representation of ECG signals [73], which share many common features with the EEG form of brain data. Furthermore, Gacek recently discussed the granular representation of time series with a number of representation alternatives and the question of forming adjustable temporal slices, and presented an optimization criterion of a sum of volumes of IG [74]. Meher and Pal presented a new rough-wavelet granular space-based model for land cover classification of multispectral remote sensing image [75], which can be used for reference to analyze 2D brain image data.

**Fig. 8:**
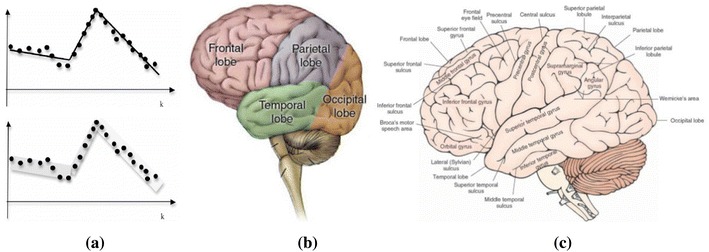
Granularities in brain big data. **a** Linear models and granular models of time series [74] and **b** lobes of human brain (coarser granularity)—http://www.brightfocus.org/alzheimers/about/understanding/anatomy-of-the-brain.html and **c** gyrus and sulcus of human brain (finer granularity)—http://what-when-how.com/neuroscience/overview-of-the-central-nervous-system-gross-anatomy-of-the-brain-part-1/

The three mechanisms of MGrC have great potentials for brain BDP in three facets. Firstly, the brain big data is of multi-granular in nature. As shown in Fig. 8a, EEG signals can be granulated like other kinds of time series [74] and be processed subsequently. For 2D and 3D brain data, they can be viewed hierarchically considering two factors. One is the organizational granularity of brain, e.g., we could view the brain in this hierarchy: whole brain → lobes → gyrus and sulcus → neurons, with lobes, gyrus and sulus shown in Fig. 8b, c, respectively. The other is the measurement granularity of activation degrees in particular region of the subject’s brain, e.g., we could granulate the activation degrees as follows: strongest → very strong → strong → weak → very weak.

Secondly, the computation performed on brain big data needs to be multi-granular and produce results of variable precision. As previously mentioned, the targets for brain BDP could be described with words; thus, they are of multi-granularity. For example, researches on the cure of a kind of brain disease may focus on the changes of certain gyrus and sulcus, while another kind of brain disease needs the neurons of temporal lobe to be investigated. Therefore, granularity optimization mechanism is useful for the former disease and granularity conversion is useful for the latter. And if the brain disease is the result of multiple causes, then multi-granularity joint computation may be required.

Thirdly, identify the proof or signs of the granular thinking in human brain, and offer valuable inspiration to computing technologies. The existence of granular thinking of human beings is already a common sense shared by the cognition and computing society, but to our best knowledge, the process of granularity optimization, granularity conversion, or MGrJC in human thinking has not been explicitly depicted by the equipments of fMRI, EEG, MEG, etc. Therefore, many details of the granular thinking in human brain still remain unknown. Using MGrC to identify and interpret the MGrC occurring in human brain is meaningful for future work.

## Conclusion

In this paper, we firstly review data space, data science, and researches on BDP, and talk about the source, form, significance, and research works of brain big data. We propose the three mechanisms of MGrC and discuss their relationship with five major models of MGrC, i.e., fuzzy set, rough set, quotient space, cloud model, and deep learning. We also discussed the key issues of current BDP and the reasons why MGrC can tackle them. Then we propose the potential of exploring brain big data with MGrC. Future research may include representing the brain big data from real world with MGrR and conducting intelligent computation based on it to offer *effective solution* to the problems to do successful research in brain BDP.
